# Fellowships, Grants, & Awards

**Published:** 2007-08

## Cancer Prevention Research Small Grant Program (R03)

This program is designed to aid and facilitate the growth of a cadre of scientists with expertise in cancer prevention research. Small grants are short-term awards that provide support for pilot projects, development and testing of new methodologies, or innovative projects that provide a basis for more extended research.

The NCI Division of Cancer Prevention invites applications that address developmental research in chemoprevention agent development, biomarkers, early detection, and nutrition science, in addition to clinical studies that focus on specific target organs. This scientific initiative (funding opportunity) is not intended for the support of research grant applications that are focused on treatment, etiology, and/or treatment-related general quality of life studies that are population based. The specific areas of research may include, but are not limited to:

### Early detection

1) identification, development, and evaluation of biological analytic techniques, methodologies, and clinical technologies relevant to preclinical cancer detection and prevention of primary and recurrent cancers; 2) cellular, molecular, and genetic tumor markers; 3) molecular epidemiology and genetic risk factors; 4) imaging technology; 5) identification of molecular signatures of cancer-associated infectious agents for earlier cancer detection and risk assessment; 6) transfer of basic laboratory findings into applications for early detection with the goal of extending this research to comparative clinical trials; 7) development and evaluation of new high-throughput genomic- and proteomic-based detection techniques as well as of measures of sensitivity, specificity, validity, and safety; 8) performance of translational research to facilitate the transfer of new technologies (to the clinical setting) for earlier detection, prevention, and risk assessment; 9) development and evaluation of computer-based data monitoring systems for analysis and interpretation of laboratory data on multiple markers of and for the development of modeling systems based on molecular, genetic, and other risk factors to be used in the evaluation of cancer prevention approaches; 10) definition and evaluation of prognostic factors of precancerous lesions by computer models that include neural networks, artificial intelligence, and other techniques; and 11) development of analytic techniques to identify populations that may be at increased risk as indicated by genetic and metabolic phenotypes (susceptibility markers).

### Chemoprevention

1) pilot testing and development of new methods of chemoprevention, dietary, or nutrition intervention; 2) development and evaluation of molecular targets to prevent, reverse, or retard progression of precancerous lesions (and hence the cancer process) by natural, synthetic, chemopreventive agents; 3) feasibility and efficacy testing of rapid screening methods to identify and prioritize new chemopreventive agents; 4) testing of new strategies to prevent cancer or its progression in persons at increased genetic risk; 5) development of innovative animal models to mimic the human cancer process in order to expedite research in cancer prevention; and 6) investigation of mechanisms of action of chemopreventive agents.

### Nutrition

1) improvements in methodology development for assessing nutritional status, metabolic patterns, and dietary modulation of genetic expression; 2) identification, development, and validation of biochemical or biological markers for measuring and monitoring dietary compliance and exposure; 3) development of reliable methods for analysis of nutrients, other dietary components, and their metabolites in foods, body fluids, and tissues; 4) development of mechanistic studies of dietary constituent interactions, gene–nutrient interactions, and dietary environmental factor interactions; 5) identification and evaluation of molecular targets to prevent, reverse, or retard progression of precancerous lesions (and hence the cancer process) by dietary/nutritional interventions; 6) determination of bioavailability and dose response of foods, nutrients, and other naturally occurring food constituents; and 7) although the specific study proposed may attempt only to obtain preliminary data and/or conduct pilot studies in support of a future, more detailed clinical study, it is important that a long-term human cancer prevention hypothesis and supporting scientific justification be presented.

This program is designed to increase the basic and applied scientific knowledge of cancer prevention research, and to enhance community-based clinical research in cancer prevention.

The common characteristic of the NIH R03 small grant is provision of limited funding for a short period of time. Examples of the types of projects that NIH ICs support with the R03 include the following: 1) pilot or feasibility studies; 2) secondary analysis of existing data; 3) small, self-contained research projects; 4) development of research methodology; 5) development of new research technology; 6) nature of the research opportunity; 7) pertinent background information that establishes the need for the research; 8) scientific knowledge to be achieved through research supported by the special program; 9) objectives of this research program; and 10) identification of the types of research and experimental approaches being sought to achieve the objectives.

This Funding Opportunity Announcement (FOA) will use the NIH Small Grants Program (R03) award mechanism. As an applicant, you will be solely responsible for planning, directing, and executing the proposed project. The total budget may not exceed $100,000 in direct costs for the entire project. The direct costs in any one year must not exceed $50,000. Please note that facilities and administrative [F&A] costs requested by any consortium participants are excluded from the direct cost limit per NIH Guide Notice NOT-OD-04-040 (http://grants.nih.gov/grants/guide/notice-files/NOT-OD-04-040.html).

The total project period for an application submitted in response to this program announcement may not exceed 2 years. NIH policy limits the number of amendments that may be submitted to two. The small grant is not renewable.

This FOA uses just-in-time concepts. It also uses the modular budget formats (see the “Modular Applications and Awards” section of the NIH Grants Policy Statement). Specifically, if you are submitting an application with direct costs in each year of $250,000 or less (excluding consortium Facilities and Administrative [F&A] costs), use the PHS398 Modular Budget component provided in the SF424 (R&R) Application Package and SF424 (R&R) Application Guide (see specifically Section 5.4, “Modular Budget Component,” of the Application Guide).

Applicants must download the SF424 (R&R) application forms and SF424 (R&R) Application Guide for this FOA through Grants.gov/Apply.

Note: Only the forms package directly attached to a specific FOA can be used. You will not be able to use any other SF424 (R&R) forms (e.g., sample forms, forms from another FOA), although some of the "Attachment" files may be useable for more than one FOA.

For further assistance, contact GrantsInfo, 301-435-0714, (telecommunications for the hearing impaired: TTY 301-451-0088), or by e-mail: GrantsInfo@nih.gov.

The application submission dates for this PAR are 21 July 2007, and 20 December 2007. Contacts: The complete list of agency contacts is available at http://grants.nih.gov/grants/guide/pa-files/PAR-06-313.

Reference: PAR-06-313.

## Translational Research for the Prevention and Control of Diabetes and Obesity (R18)

Several large, controlled clinical trials have established "gold standard" approaches for treating type 1 and type 2 diabetes, and for preventing or delaying type 2 diabetes in individuals at high risk for developing the disorder. Research is needed to translate the results of these trials into widespread practice. Studies to develop effective, sustainable, and cost-effective methods to prevent and treat diabetes and obesity in clinical health care practice and other real-world settings are appropriate targets for translational research.

The Diabetes Control and Complications Trial (DCCT), for type 1 diabetes, and the United Kingdom Prospective Diabetes Study (UKDPS), for type 2 diabetes, established the importance of intensive glycemic control in dramatically reducing the devastating complications of diabetes.

Unfortunately, the therapies proven to delay or prevent complications in these studies have not been widely incorporated into general health care practice. Prevention and treatment of long-term micro- and macrovascular complications remain a critical problem in the management of type 1 and type 2 diabetes mellitus. In the United States, diabetes is the leading cause of new blindness in working-age adults, of new cases of end-stage renal disease, and of nontraumatic lower leg amputations. In addition, cardiovascular complications are now the leading cause of diabetes-related morbidity and mortality, particularly among women and the elderly. In adults with diabetes, the risk of cardiovascular disease (CVD) is 2- to 4-fold greater than in nondiabetics. Comorbid conditions (hypertension, dyslipidemia, and smoking) combine with hyperglycemia to contribute to accelerated atherosclerosis. Clinical trial data have established the unequivocal benefit of rigorous control of glycemia and blood pressure in preventing both micro- and macrovascular complications of diabetes. Smoking cessation, aspirin therapy, and lipid control have also been shown to prevent morbidity. Despite clear-cut evidence of benefit, recently available data demonstrate that patients with diabetes are not achieving recommended levels of glycemic, blood pressure, or lipid control, or adherence to other accepted treatment guidelines.

The difficulties inherent in achieving good glucose control and preventing diabetes complications make prevention a compelling strategy. This is particularly true for type 2 diabetes, which is clearly linked to modifiable risk factors, e.g., overweight or obesity and a sedentary lifestyle. The Diabetes Prevention Program (DPP) tested strategies to prevent or delay the development of type 2 diabetes in individuals at high risk for its development by virtue of their having impaired glucose tolerance (IGT). The DPP demonstrated that intensified lifestyle or drug intervention in individuals with IGT prevented or delayed the onset of type 2 diabetes. Lifestyle intervention, leading to moderate weight loss and increased exercise, reduced diabetes incidence by 58% and the drug metformin by 31% compared with placebo. The effects were similar for men and women and for all racial and ethnic groups. Similar effects of lifestyle intervention were seen in another study conducted in Finland. Cost-effective strategies for promoting lifestyle modification leading to weight loss in these high-risk individuals, outside the setting of a controlled, clinical trial, need to be established. In addition, while behavioral treatment of obesity in adults leads to clinically significant weight loss, prevention of weight regain remains an elusive goal for many.

Overweight in childhood, the prevalence of which has more than doubled in the past 2 decades, is a major risk factor for type 2 diabetes. Indeed, the increase in overweight children has been linked to a rise in type 2 diabetes in the pediatric population. Family-based behavioral interventions have been shown to have a long-term impact on degree of overweight. However, cost-effective interventions in primary care and community-based settings are needed.

One-third of people with type 2 diabetes are undiagnosed. In addition a significant proportion of patients are diagnosed with diabetes only when they present with diabetic retinopathy or neuropathy. Epidemiologic data have shown that the risk for complications increases with duration of diabetes. Thus early diagnosis and treatment likely decreases the risk for complications. Population-based as well as generalizable, clinic-based strategies are needed to establish cost-effective programs to identify individuals at risk for diabetes or who have diabetes who could benefit from prevention or treatment programs.

The National Institute of Diabetes and Digestive and Kidney Diseases (NIDDK) through this FOA seeks to foster the development of cost effective and sustainable translational research studies to prevent and treat obesity and diabetes. The interventions designed should have the potential to be disseminated to clinical practice including individuals or communities at risk. It is not the intent of this FOA to support the development of initial efficacy trials. Rather, it is for the translation of interventions that have previously been shown to be efficacious in the research setting. Proposed studies must address issues of sustainability, cost-effectiveness, and dissemination. Interventions should be as close to cost-neutral as possible. Studies addressing minority populations at disproportionate risk for obesity, diabetes, and diabetes complications are encouraged. These proposals should focus on novel approaches to health care delivery and diabetes prevention. Proposals in which the main focus is on development and validation of culturally appropriate materials are not considered responsive.

Study design and its accompanying analysis plan must be linked to the research question. The general goal is to select a design that maximizes generalizability and minimizes bias. Relevant topics include, but are not limited to: 1) strategies to enhance glycemic control and reduce risk factors for the development of the complications of type 1 or type 2 diabetes such as blood pressure and lipids; 2) strategies to promote the adoption of healthy lifestyles which will reduce obesity and diabetes; 3) strategies for less burdensome and more cost-effective methods to identify those with or at risk of prediabetes and/or type 2 diabetes; 4) studies that test interventions to enhance long-term maintenance of weight loss and prevention of weight regain after weight loss; 5) studies to test approaches for cost-effective delivery of diabetes education and self-management instruction for improvement of glycemia; 6) studies that test interventions to treat childhood and adolescent overweight in primary care or community settings; 7) strategies to overcome health care system barriers that reduce the efficiency or effectiveness of patient/provider interaction and health outcomes; 8) strategies to promote the adoption of healthy lifestyles in women with or at risk for the development of GDM; 9) studies of interventions in work place settings or managed care organizations.

Of particular interest are studies to improve self-management and enhance health care delivery to underserved and minority populations. Such studies may seek to improve outcomes in populations (with either type 1 or type 2 diabetes) that historically have had poor glycemic, blood pressure, and other risk factor control, or promote effective prevention strategies in minority populations known to be at high risk for the development of type 2 diabetes and/or its complications.

Applicants who have received awards in response to PAR-06-358 (Planning Grants for Translational Research for the Prevention and Control of Diabetes and Obesity) should clearly identify how the Planning Grant–generated pilot and feasibility data has led to and supports the full-scale R18 proposal. Investigators who require a planning and pilot data collection phase should utilize PAR-06-358 prior to submission of an R18 proposal. All applicants should provide the rationale for the large-scale intervention and provide a full description of the setting for delivery of the intervention, primary and secondary outcomes to be assessed, the duration of follow-up, and the statistical analysis to be employed. Investigators must also address cost-effectiveness and sustainability of the proposed study design. For the project to be supported under the R18, applicants should also provide a detailed description of the target population to be studied, with justification, including a definition of the cohort by age, sex, and race/ethnicity. The applicant’s experience in recruiting this target population and the methods to be used should be described. Sample size needs required and assumptions made to estimate an appropriate sample size should be detailed including the analysis plan to be used. Applicants must state their plans for reporting accrual by sex, race, and ethnicity and for the reporting of results that examine differences in treatment effects across these subgroups (see "Inclusion of Women and Minorities in Research Involving Human Subjects"). Methods for assuring privacy and maintaining confidentiality should be included. A data and safety monitoring plan must be included.

Studies may utilize methodology from the fields of biomedical, social, or behavioral sciences, epidemiology, clinical trials, and health services and dissemination research. The primary outcome should include glycemia or weight. An intervention aimed at producing a behavioral change should be grounded in behavior change theory, which should be incorporated into the intervention. The application will be strengthened by the inclusion of a process evaluation, i.e., an evaluation of whether the intervention is actually delivered as intended. It is also recommended that applicants review the contents of the translational research meeting report URL at http://www.niddk.nih.gov/fund/other/Diabetes-Translation/conf-publication.pdf.

Investigators should provide detailed evidence that the research team has the experience and expertise to conduct the research study. Most translational research will require a multidisciplinary research team. Thus, a variety of researchers may be required for these studies, including, but not limited to, endocrinologists, public health physicians, primary care physicians, epidemiologists, statisticians, psychologists, health educators, sociologists, nurses, nutritionists, and other health-related professionals. The interdisciplinary nature of the research team should be fully described and justified.

Brief descriptions, as appropriate, of the process for biologic sample collection, storage, and handling; the laboratory tests that are needed; physical facilities, data management, and computer resources, and facilities for data retrieval and storage; and a plan for randomization of patients or settings for delivery of interventions into protocols should be provided.

Investigators located at existing Diabetes Research and Training Centers (DRTC) or proposing to collaborate with a DRTC should include a complete description of how the proposal in response to this PAR will utilize the core facilities funded through the DRTC. Investigators who are not directly affiliated with a DRTC may, if feasible, form collaborations with such centers to utilize the core resources. A list of DRTCs can be found at http://www.niddk.nih.gov/fund/other/centers.htm.

Investigators located at existing CDC-DDT supported "Translating Research into Action for Diabetes (TRIAD)" sites should include a full description of how the TRIAD sites will be advantageously utilized. TRIAD investigators should also describe how they will integrate the TRIAD sites and cohort without adversely affecting or overlapping the current and future multicenter collaborative goals of the TRIAD Study (e.g., primary hypotheses, cohort follow-up). The testing of interventions to prevent or treat disease among individuals from the TRIAD cohort is encouraged.

Interaction is encouraged between NIH-funded investigators and investigators at CDC Prevention Research Centers, a national network of 28 academic research centers that engage communities as participants in research on preventing chronic diseases. Information about CDC prevention research centers may be found at http://www.cdc.gov/prc/index.htm. In addition, applicants may be interested in the messages and resources already developed by the National Diabetes Advisory Board (NDEP) and available on the NDEP web site at http://www.ndep.nih.gov/.

This FOA will use the NIH Research Demonstration and Dissemination Project (R18) award mechanism.

The applicant will be solely responsible for planning, directing, and executing the proposed project.

This FOA uses “Just-in-Time” information concepts. It also uses the nonmodular budget formats.

Applicants must download the SF424 (R&R) application forms and the SF424 (R&R) Application Guide for this FOA through Grants.gov/Apply.

Note: Only the forms package directly attached to a specific FOA can be used. You will not be able to use any other SF424 (R&R) forms (e.g., sample forms, forms from another FOA), although some of the "Attachment" files may be useable for more than one FOA.

For further assistance, contact GrantsInfo: 301-435-0714, (telecommunications for the hearing impaired: TTY 301-451-0088) or by e-mail: GrantsInfo@nih.gov.

The application submission dates for this PA are available at http://grants.nih.gov/grants/funding/submissionschedule.htm.

The complete version of this PAR is available at http://grants.nih.gov/grants/guide/pa-files/PAR-06-457.html.

Contacts: The complete list of agency contacts is available at http://grants.nih.gov/grants/guide/pa-files/PAR-06-457.

Reference: PAR-06-457.

